# Synthesis and Characterization of Polyfumarateurethane Nanoparticles for Sustained Release of Bupivacaine

**DOI:** 10.3390/pharmaceutics12030281

**Published:** 2020-03-21

**Authors:** Soo-Yong Park, Jiin Kang, Ji-Young Yoon, Ildoo Chung

**Affiliations:** 1 Department of Polymer Science and Engineering, Pusan National University, Busan 46241, Korea; 2 Department of Dental Anesthesia and Pain Medicine, School of Dentistry, Pusan National University, Gyeongsangnam-do 50612, Korea

**Keywords:** biodegradable, polyfumarateurethane, double emulsion technique, bupivacaine

## Abstract

Biodegradable polyfumarateurethane (PFU) for use as a bupivacaine delivery vehicle, synthesized using di-(2-hydroxypropyl fumarate) (DHPF), polyethylene glycol (PEG) and 1,6-hexamethylene diisocyanate (HMDI), was designed to be degradable through the hydrolysis and enzymatic degradation of the ester bonds in its polymer backbone. Using a water-in-oil-in-water double emulsion techniques, nanoparticles encapsulating water or fluorescein isothiocyanate (FITC) were fabricated to avoid the immune system owing to the presence of PEG on their surface. The morphologies of these nanoparticles were characterized by DLS, TEM, FE-SEM, and fluorescent microscopies. The present study explored the encapsulation, loading efficiency and in vitro drug release of bupivacaine encapsulated with biodegradable PFU nanoparticles for the treatment of local anesthesia. Various concentrations of bupivacaine were encapsulated into nanoparticles and their encapsulation efficiencies and drug loading were investigated. Encapsulation efficiency was highest when 2.5% bupivacaine was encapsulated. Drug release behavior from the bupivacaine-loaded PFU nanoparticles followed a sustained release profile.

## 1. Introduction

Compared with other local anesthetic drugs that prevent pain, such as lidocaine, tetracaine, chloroprocaine, and procaine, bupivacaine provides stronger anesthesia for a longer duration; however, there is a risk of cardiac arrest during local anesthesia through intravenous injection with bupivacaine [1,2,3]. The longest anesthetic effects of bupivacaine last approximately half a day, and the dose of this drug must be sustained for longer surgeries [4,5].This continuous injection leads to an eventual increase in medical cost. Thus, many efforts have been made to develop a self-controlled analgesic system that can treat pain locally over a few days or longer periods. Several controlled drug release systems, such as biodegradable microspheres, injectable gels and patches, have been studied and evaluated [6,7,8,9]. There are two important factors to consider while designing a good drug delivery system for local anesthesia: 1) the drugs should be properly transported to maintain therapeutic focus for the maximum time; and 2) to reduce the systemic concentration of the drug and the concentration of the drug at the site of injection, the carrier/drug release should be reduced to a minimum [10]. These principles increase the therapeutic efficacy, decrease the side effects, greatly reduce medical expenses, and improve patient quality of life. Therefore, a drug delivery vehicle for bupivacaine was studied here, to increase the duration of anesthesia by controlling the release of the drug.

Segmented polyurethane elastomers have been used as biomaterials for several decades owing to their unique physicomechanical properties and favorable biocompatibility. Polyurethanes have been used in implantable devices, such as ventricular assist devices, vascular prostheses, breast implant coatings, insulators for cardiac pacing leads, tissue adhesive, skin wound dressings and so on [11,12,13]. Many current medical applications require the use of biostable polyurethanes, especially focusing on nondegradable polyurethane. However, many transplant devices, such as cardiovascular implants, artificial skin, cancellous bone graft substitutes and nerve conduits, would have advantages from being prepared using elastomeric biodegradable polyurethanes. The degradation mechanisms of these polyurethanes can be generally classified as hydrolytic, oxidative and enzymatic. Hydrolytically degradable polyurethanes have been synthesized by the incorporation of soft segments such as polyhydroxyamide and polyesters [14].

Biodegradable polymer was designed to have an ester group on its backbone to allow its degradation into biocompatible diacids and diols [15,16,17,18,19,20]. We also previously reported a number of studies on the synthesis of biodegradable polymers based on aliphatic polyester, polyanhydride, poly(amino acid), biopolymer and their in vitro and in vivo cytotoxicities—after antitumor, antibacterial, gene-delivered nanoparticles were fabricated [21,22,23,24,25,26,27].

Biodegradable polyurethane may also be synthesized by the reaction of poly(ethylene glycol) (PEG) and hexamethylene diisocyanate (HMDI). PEG as soft segments can make polyurethane susceptible to degradation by oxidation [28]. PEG on the surface of nanoparticles prepared by double emulsion method helps to avoid the immune system such as protein absorption. Because the size of nanoparticles plays a major role in biocompatibility and effect of drug release, bupivacaine-loaded nanoparticles were studied [29,30].

In this study, polyfumarateurethane (PFU) was designed to be degraded through hydrolysis and enzymatic degradation mechanisms due to the presence of ester groups derived from di-(2-hydroxypropyl fumarate) (DHPF) in its backbone, and used to fabricate biodegradable nanoparticles for sustained release of bupivacaine. DHPF was used not only to increase the chain length but also to improve biodegradability of PFU. The PEG in the PFU backbone is a soft segment macrodiol that minimizes the toxicity of polyurethanes [31,32].

Methoxy-polyethylene glycol-b-polylactide (mPEG-b-PLA) copolymer was also synthesized by ring-opening polymerization and used as a surfactant in double emulsion, and endowed produced nanoparticles with enhanced biocompatibility [33,34,35]. The double emulsion method has been reported to be suitable for the encapsulation of hydrophilic drugs; consequently, it has potential for use in the injection of bupivacaine [36,37,38]. During double emulsion formation, the mPEG segment of the block copolymer stabilizes the interfacial tension of the nanoparticles owing to its amphipathic nature, and its presence on the surface obscures particles from detection by the immune system [39,40,41,42].

## 2. Materials and Methods

### 2.1. Materials

Fumaryl chloride, polyethylene glycol (PEG, Mw 2000), l-lactide, tin(II) 2-ethylhexanoate (stannous octoate), fluorescein isothiocyanate (FITC) and polyvinyl alcohol (PVA, Mw 30,000-70,000) were purchased from Sigma Aldrich (Milwaukee, WI, USA). 1,2-Propanediol, dibutyltin dilaurate, and methoxy-polyethylene glycol (mPEG, Mw ~2000) were purchased from Tokyo Chemical Industry (TCI) (Tokyo, Japan). Potassium carbonate, ethyl acetate, hexamethylene diisocyanate (HMDI), dimethylformamide, toluene, and chloroform were purchased from Daejung Chemical & Metal (Siheung, Korea). For the purification of PEG, toluene and PEG were added to a single-neck round-bottom flask, and water was removed by azeotropic distillation.

### 2.2. Synthesis of PFU

Di-(2-hydroxypropyl fumarate) (DHPF) was synthesized according to our previous method [43]. Briefly, a solution of fumaryl chloride in ethyl acetate was added dropwise to a solution of ethyl acetate containing propylene glycol at 0 °C for 2 h under nitrogen in the presence of potassium carbonate which is a proton scavenger. The mixture was allowed to react for an additional 3–4 h at room temperature with continuous stirring. After the reaction was completed, the reaction solution was washed with water and brine. The organic layer was collected and dried with magnesium sulfate. After filtration and evaporation of the solvent, DHPF was obtained as a yellowish viscous liquid.

**Scheme 1:**
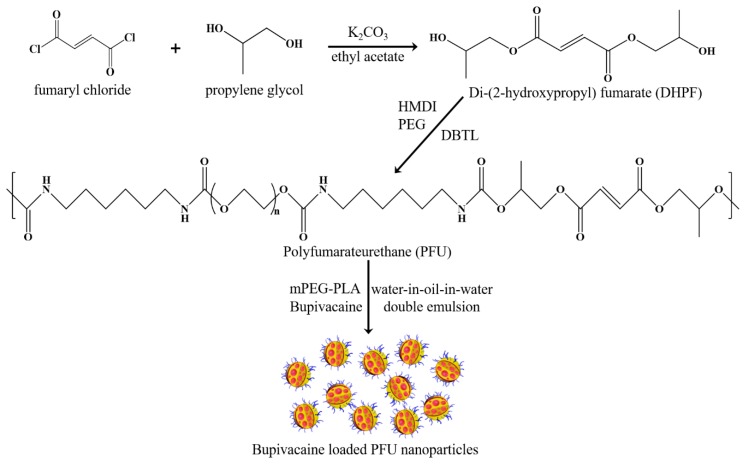
Fabrication of biodegradable bupivacaine-loaded polyfumarateurethane (PFU) nanoparticles.

To a solution of PEG (0.002 mol) in anhydrous DMF (40 mL) at 70 °C, HMDI (0.004 mol) and 10 µL of DBTL were added with nitrogen purging. Both chemicals were diluted with DMF before injecting to above solution. After the reaction mixture was stirred for 2 h, 0.002 mol of diluted DHPF was added to the reactor and the reaction was further continued for 4 h at the same temperature. Then, the solution was precipitated in a cold NaCl solution (12 g/L) with stirring of glass rod. After quenching for 30 min in the refrigerator, PFU was obtained by centrifugation. Further centrifugation with distilled water was conducted to wash the product. Final product was gained through lyophilization for 2 days (Scheme 1).

### 2.3. Synthesis of mPEG-PLA

To a solution of mPEG (0.8 mmol) and L-lactide (16.64 mmol) in dried toluene (120 mL), stannous octoate (100 mg in 100 mg/mL toluene) was added. The reaction mixture was heated to 130 °C under a nitrogen atmosphere. After the reaction was continued for 12 h, the reaction mixture was cooled to room temperature and toluene was completely removed under reduced pressure. The resulting white viscous compound was dissolved in 9 mL of dichloromethane and precipitated into 100 mL of cold ether. The precipitate was collected and dried under vacuum at room temperature for 2 days to obtain mPEG-b-PLA as a white solid.

### 2.4. Fabrication of Blank and FITC-Loaded PFU Nanoparticles

As shown in Table 1, blank and FITC-loaded PFU nanoparticles were fabricated using water-in-oil-in-water double emulsion techniques using FITC solution, or distilled water, chloroform, and 5% PVA solution. FITC was encapsulated to confirm the structure of nanoparticles with loading of drug in water-in-oil-in-water double emulsion processes. FITC (0.1 mg) was dissolved in distilled water (1 mL) for preparing FITC PFU nanoparticles and distilled water (1 mL) without FITC was used for preparing blank nanoparticles. Blank nanoparticles, which encapsulate distilled water as the first water phase, were analyzed before drug loading. In a round-bottomed flask, after PFU (198 mg) in 8 mL of chloroform and mPEG-PLA (2 mg) in 2 mL of chloroform were prepared, both PFU and mPEG-PLA solutions and a solution of distilled water or FITC were added in sequence. After stirring at 2000 rpm for 1 min, the stirring rate was decreased to 1600 rpm and then continuously stirred for 3 min with the addition of 5% PVA solution to form double emulsion nanoparticles. After the remained chloroform was removed by air-drying overnight, centrifugation was carried out at 10,000 rpm for 10 min at 4 °C to remove the impurities such as PVA and free FITC. After the crude product was collected, re-dispersed in distilled water and centrifuged again three times, it was lyophilized to obtain pure biodegradable PFU nanoparticles.

### 2.5. Fabrication of Bupivacaine-Loaded PFU Nanoparticles and Their Release Characteristics

All the procedures are same as FITC PFU nanoparticles preparation except for using bupivacaine solution instead of FITC solution. Bupivacaine concentrations of 2.5%, 5%, 7.5% and 10% were encapsulated to nanoparticles for evaluating the optimized bupivacaine-loading percentage. Each weight of bupivacaine was dissolved in 1 mL of distilled water. The particles were evaluated to verify the optimized bupivacaine quantity used relative to the polymer quantity used. The samples were gently stirred in chloroform and distilled water. Water layer was collected, and the concentration of bupivacaine was determined by UV Vis spectrometer. Different concentrations of bupivacaine-loaded nanoparticles were suspended in phosphate buffer saline (PBS) and monitored for 1, 2, 4, 7 and 14 days. The cumulative release from the extract was measured in the supernatant after centrifugation.

### 2.6. Measurements

The chemical structures were characterized by Nuclear Magnetic Resonance (^1^H NMR, FT-300MHz Varian Gemini 2000 spectrophotometer, Palo Alto, CA, USA) and Fourier transform-infrared (FT-IR, Agilent Technologies Cary 600 Series, Santa Clara, CA, USA) spectroscopies. ^1^H NMR and FT-IR spectroscopies were performed three times with each 256 and 128 scans. The size of nanoparticles was characterized by Dynamic Light Scattering (DLS, Zetasizer Nano-S90, Malvern Panalytical Ltd, Malvern, UK) equipped with a 633 nm He-Ne laser by dispersing nanoparticles in water. Drug encapsulation, loading efficiency and cumulative drug release were identified by Ultraviolet-visible spectrometer (UV–Vis, Jasco V-630, Jasco, Easton, MD, USA) ranged from 200 to 400 nm. The wavelength maximum was selected at 263 nm to quantify the concentration of bupivacaine with correlation coefficient of 0.9998 for bupivacaine.

The nanoparticles were also examined by transmission electron microscopy (TEM, JEOL JEM-1400, Tokyo, Japan), and field emission-scanning electron microscopy (FE-SEM, Carl Zeiss AG SUPRA25, Oberkochen, Germany). Double emulsion structures of nanoparticles were confirmed by fluorescence microscopy (Meiji Techno MX-6300, Saitama, Japan). The results of FE-SEM, TEM, and fluorescence microscopy were helpful to confirm the size and morphology of encapsulated structures.

Molecular weight and polydispersity were determined by gel permeation chromatography (GPC), conducted with a Waters 1515 pump and Waters 2414 differential refractometer using Waters columns (Styrogel HR 2, HR4, HR5E) in DMF as an eluent at 35 °C and at a flow rate of 1 mL/min. Linear poly(methyl methacrylate) standards were used for calibration.

## 3. Results and Discussion

### 3.1. Characterization of DHPF, PFU, and mPEG-PLA

**Figure 1:**
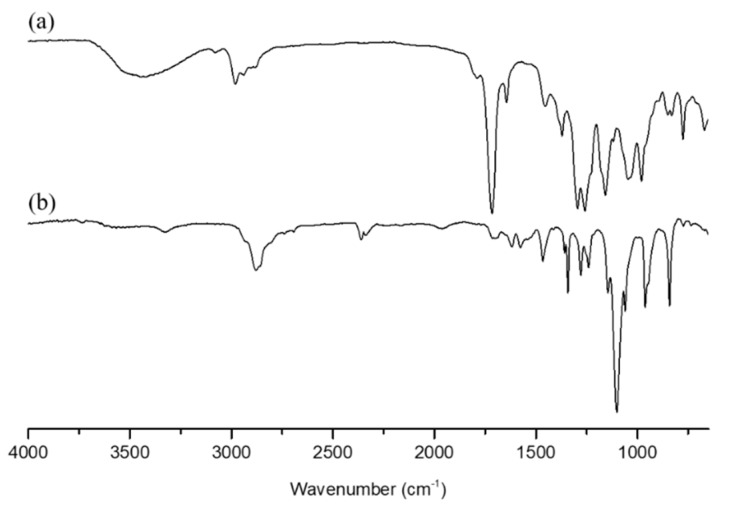
Fourier transform-infrared (FT-IR) spectra of (**a**) di-(2-hydroxypropyl fumarate) (DHPF) and (**b**) PFU.

In this study, DHPF, as a biodegradable chain extender for the polymerization of PFU, was synthesized using fumaryl chloride, propylene glycol, and potassium carbonate as a proton scavenger, and confirmed by FT-IR and ^1^H-NMR analyses. As shown in Figure 1a, DHPF exhibited strong FT-IR peaks at 3450, 1718, 1600, 1294, and 1259 cm^−1^ associated with hydroxyl, carbonyl, carbon-carbon double bond, ester I, and ester II groups, respectively. ^1^H-NMR spectrum of DHPF showed several characteristic peaks such as –CH (6.7 ppm with quartet, 3.8 ppm), –OH (4.9 ppm), –CH_2_ (4.2 ppm) and –CH_3_ (0.2 ppm).

**Figure 2:**
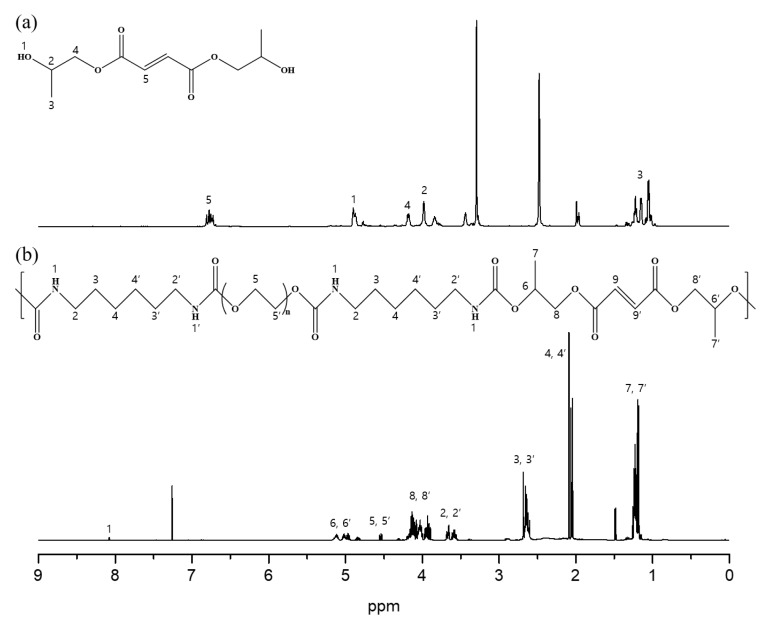
^1^H-NMR spectra of (**a**) DHPF and (**b**) PFU.

The chemical structure of PFU was also assigned by FT-IR and ^1^H-NMR spectroscopies. As seen from Figure 1b, FT-IR spectrum of PFU showed several characteristic absorption peaks arisen from urethane linkages such as amide, carbonyl groups at 3290, 1720 cm^−1^ together with the disappearance of isocyanate group at 2310 cm^−1^, which confirmed the successful PFU synthesis. The ^1^H-NMR spectrum of PFU, as shown in Figure 2b, contained the following characteristic chemical shifts: δ (ppm) = –NH (8 ppm), –CH_2_– (4.6 ppm, 3.7 ppm, 3.2 ppm, 2.0 ppm), –CH_2_ (1.3 ppm), =CH_2_ (7.1 ppm), and –CH– (4.3 ppm). GPC analysis indicated that PFU had molecular weight of 27,000 with a polydispersity of 1.71, which is an advantageous molecular weight for biodegradable nano-vehicles.

**Figure 3:**
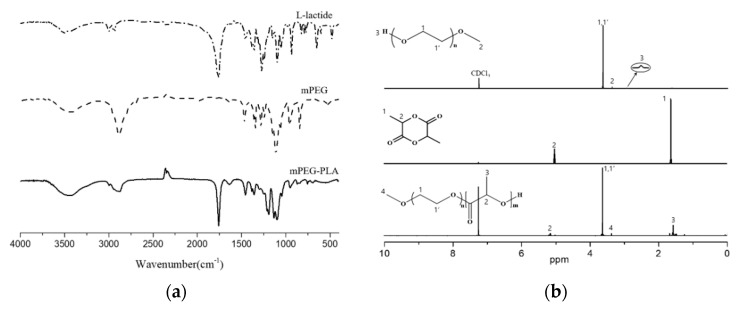
(**a**) FT-IR and (**b**) ^1^H-NMR spectra of L-lactide, mPEG and mPEG-PLA.

Prior to the use of mPEG-PLA as a biocompatible and biodegradable surfactant during the fabrication of PFU nanoparticles by double emulsion technique, mPEG-PLA block copolymer was synthesized by the ring-opening polymerization of L-lactide initiated from mPEG (2 kDa) using stannous octoate as a catalyst, and characterized by FT-IR and NMR spectroscopies. FT-IR spectra of l-lactide, mPEG, mPEG-PLA showed carbonyl group (1600 cm^−1^) of l-lactide and hydroxyl group (3500 cm^−1^) in mPEG-PLA. ^1^H-NMR spectra indicated methylene group (–CH_2_) of the repeating unit of mPEG and methyl group (–CH_3_) of l-lactide at 4.6 and 1.5 ppm, respectively. The molecular weight of this polymer was approximately 4400 (Figure 3).

### 3.2. Particle Size Distribution and Encapsulation

**Figure 4:**
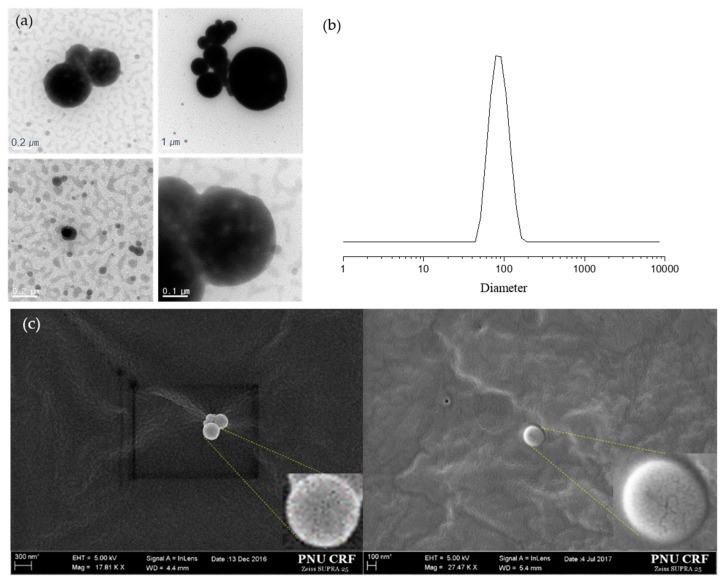
(**a**) TEM images, (**b**) DLS analysis, and (**c**) FE-SEM images of PFU nanoparticles.

The shape and size of blank nanoparticles were confirmed by DLS, TEM and FE-SEM microscopies. In DLS results, the average diameter of nanoparticles was confirmed to be approximately 100 nm. Similar results were also conveyed by TEM and FE-SEM images (Figure 4). In TEM images, the relatively brighter part in nanoparticles indicates pores in the particles. Pores and cracks made by double emulsion technique could also be confirmed in the magnified SEM images. Nanometer-scale delivery systems offer certain distinct advantages for drug delivery, as nanoparticles can penetrate deeply into tissues and are generally taken up efficiently by the cells. The synthesized nanoparticles based on PFU had a spherical morphologies with smooth surfaces, although some particles were aggregated.

**Figure 5:**
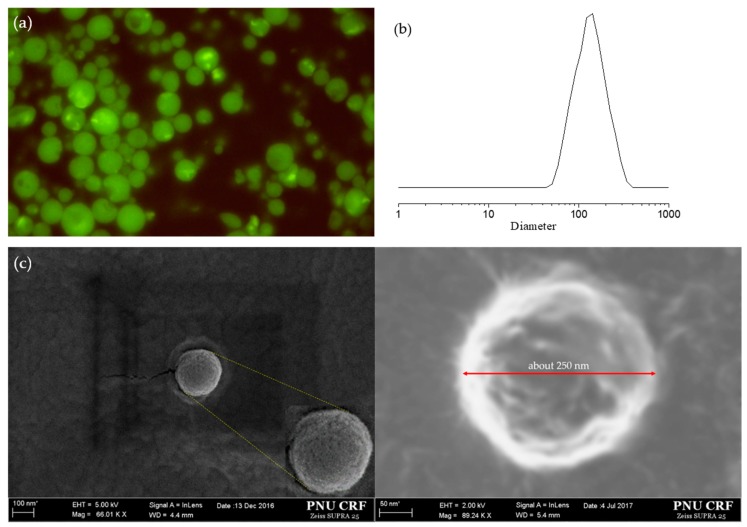
(**a**) Fluorescence microscopy, (**b**) DLS analysis, and (**c**) SEM images of FITC-BSA PFU nanoparticles.

From the fabrication of FITC-loaded PFU nanoparticles as an indicator of encapsulation, as shown in Figure 5a, it was confirmed that FITC was successfully encapsulated in PFU nanoparticles as detecting brighter spots inside of nanoparticles. FE-SEM images of these nanoparticles showed a porous and wrinkly spherical morphologies with an average diameter of approximately 250 nm. Good agreement of the average size of nanoparticles was also obtained from DLS results, which is an appropriate size for endocytosis of nanoparticles in drug delivery system (Figure 5).

### 3.3. Encapsulation and Drug Loading Efficiency

To evaluate the amount of encapsulated drug, encapsulation efficiency and the drug-loading efficiency were studied. Encapsulation efficiency refers to the amount of encapsulated drug as a percentage of the total amount of the drug used in the fabrication process; thus, a good encapsulation efficiency is a reflection of the success of the encapsulation progress. Drug loading refers to the amount of loaded drug as a percentage of the total weight of the microspheres, and this parameter indicates the amount of drug that is available for controlled release. Drug-loaded nanoparticles (5 mg) were added to 2 mL of chloroform and 2 mL of distilled water, and the mixture was gently stirred overnight to extract the drug. After the extraction was completed, the aqueous solution was assayed using UV–Vis spectrometer at a fixed λ_max_ of 263 nm in order to determine the amount of bupivacaine encapsulated. The encapsulation efficiency was calculated by using following equation.

$$\mathrm{Encapsulation}\;\mathrm{efficiency}\;(\%)=\frac{\mathrm{Weight}\;\mathrm{of}\;\mathrm{encapsulated}\;\mathrm{drug}}{\mathrm{Total}\;\mathrm{weight}\;\mathrm{of}\;\mathrm{drug}}\times 100=\frac{C_{e}\times V_{e}/W_{e}}{C_{t}\times V_{t}/W_{t}}\times 100$$

$C_{e}$: concentration of extracted drug solution
$V_{e}$: volume of aqueous buffer used for the extraction
$W_{e}$: weight of nanoparticles used for the extraction
$C_{t}$: concentration of drug solution used in the encapsulation process
$V_{t}$: volume of drug solution used in the encapsulation process
$W_{t}$: weight of total nanoparticles yielded

Using the same variables, drug loading was also calculated using following equation.

$$\mathrm{Drug}\;\mathrm{loading}\;(\%)=\frac{\mathrm{Weight}\;\mathrm{of}\;\mathrm{loaded}\;\mathrm{drug}}{\mathrm{Weight}\;\mathrm{of}\;\mathrm{nanoparticles}}\times 100=\frac{C_{e}\times V_{e}}{W_{e}}\times 100$$

$C_{e}$: concentration of the extracted drug solution
$V_{e}$: volume of aqueous buffer used for the extraction
$W_{e}$: weight of the nanoparticles used for the extraction

Five milligrams of each of the different bupivacaine-loaded nanoparticles was weighed and stored in a test tube. Then, 1.1 mL of PBS with 0.1% sodium azide was added to each tube and placed in incubator at 37 °C. After the indicated time interval (after 1, 2, 4, 7, and 14 days), the samples were centrifuged at 10,000 rpm for 10 min. One milliliter of the supernatant from each tube was collected and added to 1 mL of fresh PBS with sodium azide, and then the microtubes were placed in the incubator. Each collected supernatant was measured by UV Vis spectrometer to determine the concentration of bupivacaine.

**Figure 6:**
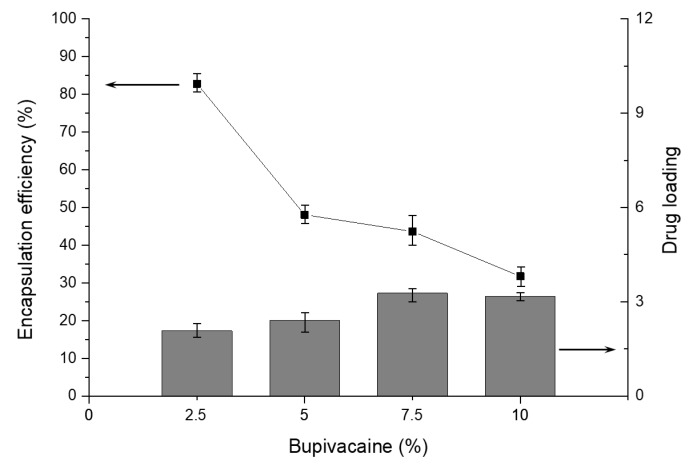
Encapsulation efficiency and drug loading of bupivacaine-loaded PFU nanoparticles. (standard deviation for *n* = 4).

Bupivacaine percentages of 2.5%, 5%, 7.5% and 10% were encapsulated into nanoparticles and encapsulation efficiency and drug loading were investigated. Encapsulation efficiency was highest when 2.5% bupivacaine was encapsulated. The encapsulation efficiency decreased as the concentration of bupivacaine increased (Figure 6).

Drug loading is generally affected by particle size, shape and composition, as well as the solubility of drug. As shown in Figure 6, as bupivacaine concentration increased, drug loading also increased with an upper limit around 3%. This phenomenon may be attributed to the fact that bupivacaine can be loaded into PFU nanoparticles through both encapsulation inside nanoparticles and physical adsorption onto the surface of nanoparticles. As mentioned above, although the limited encapsulation capacity inside nanoparticles, the higher bupivacaine concentration, the higher drug loading because of the increased drug amount on the particle surface by physical adsorption. On the other hand, encapsulation efficiency should decrease with increasing bupivacaine concentration. This is not surprising because the total amount of encapsulated bupivacaine in PFU nanoparticles always remained constant, even though bupivacaine concentration increased.

### 3.4. In Vitro Cumulative Drug Release with Stability of NPs in PBS

**Figure 7:**
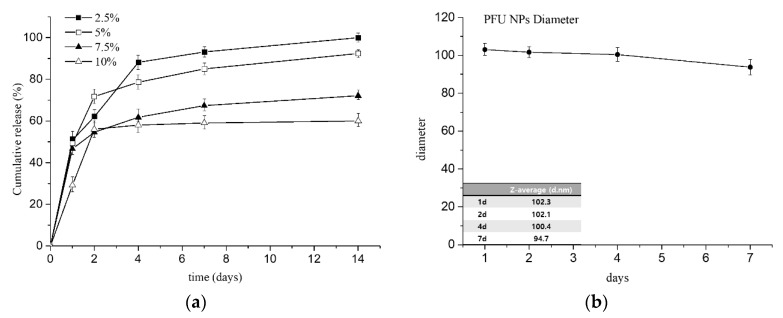
(**a**) Cumulative release profiles and (**b**) physiological stability of bupivacaine-loaded nanoparticles. (standard deviation for *n* = 4).

As shown in Figure 7a, the drug release behavior of bupivacaine-loaded PFU nanoparticles showed sustained release profile especially at low loading concentrations of bupivacaine. Except for 10% bupivacaine-loaded nanoparticles, all groups showed sustained release profiles. Due to both relatively higher cumulative ratio and longer period release of bupivacaine than other concentrations, the nanoparticles with 2.5% of bupivacaine could be selected as the optimized drug concentration. The characteristic features such as biodegradability, encapsulation by water-in-oil-in-water double emulsion techniques, and prolonged release could increase the anesthetic effect of bupivacaine while decreasing its side effects, such as cytotoxicity and cardiac arrest. The physiological stability of these PFU nanoparticles was also evaluated by in vitro test in pH 7.4 phosphate-buffered saline (PBS). The PFU nanoparticles were suspended in PBS at 37 °C using shaking water bath and the size changes of PFU nanoparticles were continuously monitored by DLS for 7 days. The stability assay showed that PFU nanoparticles remained stable in PBS showing an acceptable size variation for up to 7 days (Figure 7b). This supports that PFU nanoparticles could undergo bulk degradation, meaning that the particle size can be maintained during degradation process, because the rate of water penetration into nanoparticles is faster than rate at which the polymer is degraded, which is commonly observed for aliphatic polyesters including polyglycolide, polylactic acid and polyfumarate [44,45,46].

## 4. Conclusions

Biodegradable and biocompatible polyfumarate-urethane (PFU) has been synthesized using PEG, HMDI and DHPF. The resulting PFU was designed to be degraded through hydrolysis and enzymatic mechanisms. PFU nanoparticles were fabricated using a double emulsion technique to allow its sustained drug release. In this process, mPEG-PLA was used for surfactant to cover nanoparticles with PEG to confer biocompatibility with the immune system. Through TEM, FE-SEM, DLS and the fluorescence microscopy measurements, the particle size and morphology of nanoparticles were characterized. Bupivacaine-loaded nanoparticles with four different encapsulation percentages were prepared and evaluated for their encapsulation efficiency, drug loading and in vitro cumulative drug release. PFU nanoparticles showed promising for potential anesthetic drug release carriers due to their capacity for prolonged sustained release. Future studies will focus on in vitro and in vivo cytotoxicities including toxicities of both nanoparticles and degradation products.

## Figures and Tables

**Table 1:** Formulations of PFU nanoparticles by double emulsion technique.

Polymer	Concn (mg/mL)	Mass (mg)	Vol (mL)	Mass % (*w/w*)	Vol % (*v/v*)
**Blank PFU Nanoparticles**					
PFU	24.8	198.0	8.0	99.0	7.2
mPEG-PLA	3.0	2.0	2.0	1.0	1.8
FITC	-	-	1.0	-	0.9
5% PVA	-	-	100.0	-	90.1
Total	-	200.0	111.0	100.0	100.0
**FITC-loaded PFU Nanoparticles**					
PFU	24.8	198.0	8.0	98.9	8.1
mPEG-PLA	3.0	2.0	2.0	1.0	0.9
FITC	0.1	0.1	1.0	0.1	0.9
5% PVA	-	-	100.0	-	90.1
total	-	200.1	111.0	100.0	100.0
